# Whole exome sequencing identifies novel candidate genes that modify chronic obstructive pulmonary disease susceptibility

**DOI:** 10.1186/s40246-015-0058-7

**Published:** 2016-01-07

**Authors:** Shannon Bruse, Michael Moreau, Yana Bromberg, Jun-Ho Jang, Nan Wang, Hongseok Ha, Maria Picchi, Yong Lin, Raymond J. Langley, Clifford Qualls, Julia Klensney-Tait, Joseph Zabner, Shuguang Leng, Jenny Mao, Steven A. Belinsky, Jinchuan Xing, Toru Nyunoya

**Affiliations:** Lovelace Respiratory Research Institute, 2425 Ridgecrest Drive SE, Albuquerque, NM 87108 USA; Department of Genetics, Rutgers, The State University of New Jersey, 145 Bevier Road, Piscataway, NJ 08854 USA; Department of Biochemistry and Microbiology, Rutgers, the State University of New Jersey, Piscataway, NJ USA; Department of Internal Medicine, University of New Mexico and New Mexico VA Health Care System, Albuquerque, NM USA; Biomedical Research Institute of New Mexico, Albuquerque, NM USA; Department of Medicine, University of Iowa, Iowa City, IA USA

**Keywords:** Whole exome sequencing, Cigarette smoke, COPD, Susceptible smokers, Resistant smokers

## Abstract

**Background:**

Chronic obstructive pulmonary disease (COPD) is characterized by an irreversible airflow limitation in response to inhalation of noxious stimuli, such as cigarette smoke. However, only 15–20 % smokers manifest COPD, suggesting a role for genetic predisposition. Although genome-wide association studies have identified common genetic variants that are associated with susceptibility to COPD, effect sizes of the identified variants are modest, as is the total heritability accounted for by these variants. In this study, an extreme phenotype exome sequencing study was combined with in vitro modeling to identify COPD candidate genes.

**Results:**

We performed whole exome sequencing of 62 highly susceptible smokers and 30 exceptionally resistant smokers to identify rare variants that may contribute to disease risk or resistance to COPD. This was a cross-sectional case-control study without therapeutic intervention or longitudinal follow-up information. We identified candidate genes based on rare variant analyses and evaluated exonic variants to pinpoint individual genes whose function was computationally established to be significantly different between susceptible and resistant smokers. Top scoring candidate genes from these analyses were further filtered by requiring that each gene be expressed in human bronchial epithelial cells (HBECs). A total of 81 candidate genes were thus selected for in vitro functional testing in cigarette smoke extract (CSE)-exposed HBECs. Using small interfering RNA (siRNA)-mediated gene silencing experiments, we showed that silencing of several candidate genes augmented CSE-induced cytotoxicity in vitro.

**Conclusions:**

Our integrative analysis through both genetic and functional approaches identified two candidate genes (*TACC2* and *MYO1E*) that augment cigarette smoke (CS)-induced cytotoxicity and, potentially, COPD susceptibility.

**Electronic supplementary material:**

The online version of this article (doi:10.1186/s40246-015-0058-7) contains supplementary material, which is available to authorized users.

## Background

Chronic obstructive pulmonary disease (COPD), characterized by a permanent airflow limitation, is a growing public health threat and a leading cause of disability and mortality worldwide [1, 2]. Although cigarette smoking is a major risk factor [3], evidence suggests that genetic factors modulate smoking-induced COPD [4]. For example, alpha1 antitrypsin deficiency (A1ATD), caused by a homozygous missense mutation of *SERPINA1* (PiZ; Glu342Lys) or compound heterozygosity of the PiZ and PiS variants, contributes to lung function decline among smokers [5, 6]. However, A1ATD accounts for only 2–3 % of COPD cases [7]. Genome-wide association study (GWAS) and fine-mapping studies have revealed common (>5 % minor allele frequency) single nucleotide polymorphisms (SNPs) that are associated with COPD in a number of candidate genes, including *CHRNA3/5*, *FAM13A*, and *HHIP* [8–11]. However, the effect sizes for these associated common variants are small relative to those of *SERPINA1*-mediated A1ATD and COPD. Sequencing studies of COPD hold the promise of identifying rare variants with large effects on disease susceptibility.

**Fig. 1:**
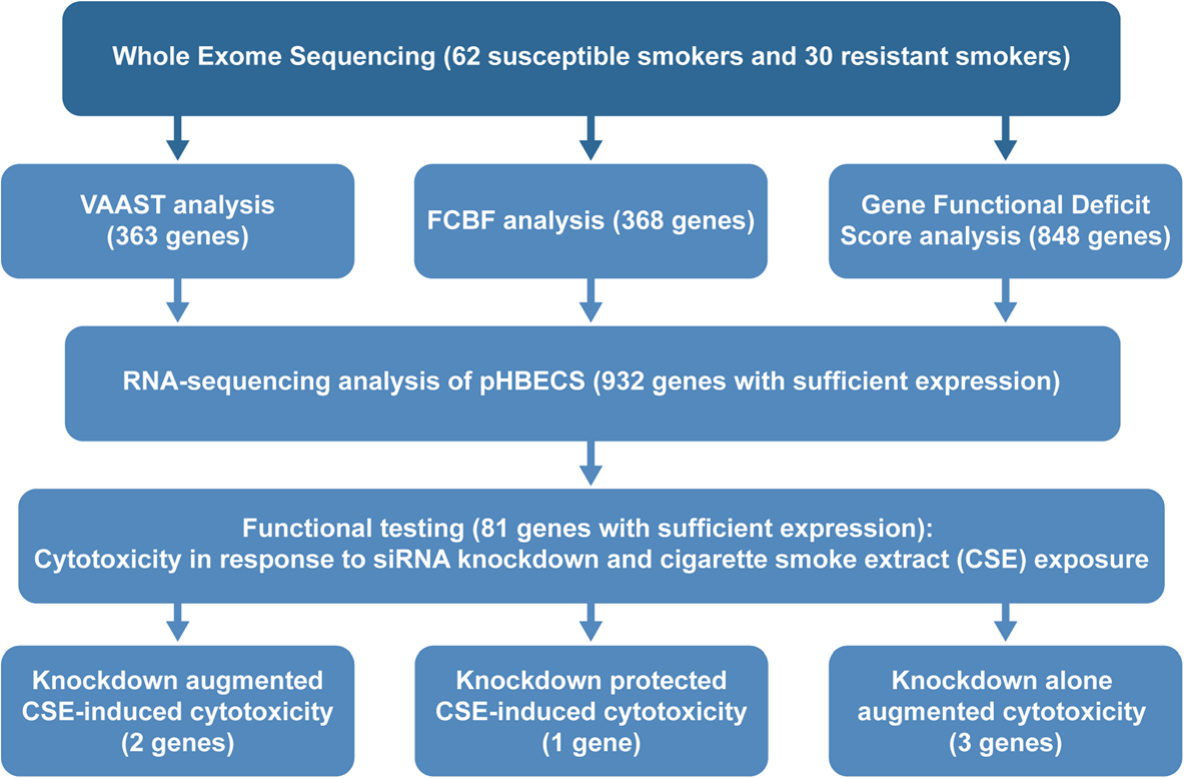
Identifying candidate COPD genes through genomic and functional approaches. WES in 62 highly susceptible smokers and 30 exceptionally resistant smokers were conducted to identify exonic variants that may contribute to disease risk or resistance to CS. Top scoring candidate genes from the rare variant and gene set analyses were further filtered by requiring that the gene be expressed in primary HBECs, and 81 candidate genes were selected for in vitro functional testing in CSE-exposed HBECs. Using siRNA-mediated gene silencing experiments, we identified candidate genes whose knockdown augmented CSE-induced cytotoxicity, protected CSE-induced cytotoxicity, or alone-reduced cell viability

Recent developments in DNA sequencing technologies have dramatically reduced the cost of acquiring genetic data. In particular, whole exome sequencing (WES) has become a valuable tool for studying both rare and common diseases [12]. WES allows interrogation of the entire coding portion of the human genome at a fraction of the cost of whole-genome sequencing. In a recent study, WES led to the identification of a non-synonymous SNP in the gene *CCDC38* that was associated with resistance to cigarette smoke (CS)-related airflow obstruction, assessed by sequencing heavy smokers with normal lung function [13]. Therefore, to identify potential causal variants for both COPD and CS resistance, we conducted WES of 62 smokers with very advanced COPD and 30 resistant smokers. We sought to sample from the extremes of the phenotypic distribution, under the assumption that this would enrich sampling of rare causal variants with large effect sizes [14]. The COPD group was thus selected to contain the youngest, lightest smoking, and most severely diseased individuals from available cohorts; the resistant group was selected to contain the oldest, heaviest smoking, and healthiest individuals (i.e., no comorbidities) with normal lung function. The primary analytical approach was aimed at identifying rare variants (and the associated genes) contributing to these two phenotypes. Additionally, we employed approaches focusing on the collective impact of multiple weakly deleterious variants (both common and rare). Candidate genes were further filtered using gene expression analysis to retain a total of 81 genes, which were functionally tested in CS-exposed, immortalized human bronchial epithelial cells (HBECs) using a gene knockdown approach (Fig.  1). This systematic multi-layered approach may help remove false-positives and prioritize true COPD (or CS-induced damage resistance) genes. HBECs were chosen as an in vitro screening model because airway epithelial cells are the primary target of CS exposure. CS exposure induces inflammation, DNA damage, and autophagy that causes lung epithelial cells to undergo various cell fates, including cell death, cellular senescence, and/or transformation [15]. Although there is no sine qua non cellular phenotype of lung epithelial cells in COPD, the lungs of patients with COPD exhibit a significant increase in apoptotic cells [16, 17]. We thus chose in vitro cell viability as an endpoint.

## Results

### VAAST analysis

We performed WES on 62 susceptible smokers with COPD and 30 resistant smokers with normal spirometries in the absence of significant comorbidities (Table 1). All individuals were of self-reported non-Hispanic white ethnicity. We used the Variant Annotation, Analysis, and Search Tool (VAAST) program to prioritize candidate genes (Additional file 1) [18, 19]. Given allele frequency and amino acid substitution severity, VAAST prioritizes genes based on their disease-causing likelihood. We performed VAAST analysis under different settings, including different modes of inheritance, penetrance models, and individual sets. From the VAAST output, several hundred genes had a *p* value <0.05 under each analysis set-up (Table 2). We did not use a more stringent cutoff or a false discovery ratio (FDR) correction for the *p* value because these candidate genes would be further prioritized in the downstream analyses and validated by an in vitro assay.

**Table 1:** Demographic data of resistant smokers and COPD subjects

	Resistant smokers	COPD	*p* value
Gender (M/F)	7/23	34/28	<0.01^*^
Age	65.7 ± 5.3	57.7 ± 7.0	<0.0001^#^
Pack year	62.2 ± 20.6	45.7 ± 19.0	0.0003^#^
FEV1/FVC%	76.9 ± 5.9	29.5 ± 9.5	<0.0001^#^
% predicted FEV	103.9 ± 11.4	25.8 ± 11.4	<0.0001^#^
% predicted DLCO	N/A	32.8 ± 16.5	N/A

^*^Fisher exact test

^#^ *t* test

**Table 2:** The number of candidate genes under different VAAST analysis categories

VAAST analysis category			Analysis	Candidate genes
Inheritance	Parameters	Samples		
Dominant	5 % background allele frequency	Resistant vs. susceptible background	1	534
		Susceptible vs. resistant background	2	757
	Complete penetrance	Resistant vs. susceptible background	3	343
		Susceptible vs. resistant background	4	366
Recessive	5 % background allele frequency	Resistant vs. susceptible background	5	532
		Susceptible vs. resistant background	6	759
	Complete penetrance	Resistant vs. susceptible background	7	195
		Susceptible vs. resistant background	8	269

Some of the top-ranking candidate genes from the VAAST analysis contain rare deleterious mutations in multiple susceptible individuals. For example, the gene *TACC2* was ranked 6th with complete penetrance and 22nd with 5 % prevalence in our VAAST analysis of the susceptible group under a recessive inheritance model. One of the susceptible individuals carries a nonsense stop-gain mutation (chr10: 123842508) in *TACC2* (a glutamine mutated to a stop codon). Six additional susceptible individuals carry novel mutations at different positions of this gene, making *TACC2* a promising candidate. Using Sanger sequencing, we validated all nonsense and non-synonymous *TACC2* variants where the DNA samples were available, for a total of seven variants in eight COPD samples, including four novel variants (Additional file 7: Table S1). The 100 % validation rate for the seven variants suggests that the variant calls are of high quality.

### Gene functional deficit score-based TTest and FCBF analysis

To determine the collective impact of all variants, including common and weakly deleterious variants, a gene functional deficit score was calculated for all genes. Of our set of 15,594 variant-affected genes in at least one individual in either the susceptible smokers or the resistant smokers, 848 genes attained *p* values of <0.05 (*TTest set*); 194 genes attained a *p* value <0.01 for the *t* test comparing resistant individuals to susceptible individuals (TTest tab in Additional file 2). However, none of the *p* values were significant after Bonferroni correction for multiple comparisons. To account for lack of variability and small numbers of samples in the score distributions, we further reduced the reference gene set by keeping only those affected in at least 10 individuals in both resistant and susceptible cohorts (8877 genes affected in at least 20 individuals). This reduction did not produce any genes with significant corrected *p* values either. This result suggests that either more individuals are necessary for improved resolution of the study or that the resistant and susceptible phenotypes are the result of additive or epistatic interaction of altered function of multiple genes.

Next, we performed fast correlation-based filter solution (FCBF) attribute analysis and the FCBF selection resulted in a set of 368 relevant genes (*FCBF set*) (of these, 59 attained an [average merit − standard error] > 0; FCBF tab in Additional file 2). Of the entire set, 62 % (229) and 38 % (139) of the genes had higher average scores in susceptible and resistant individuals respectively (FCBF tab in Additional file 2). The large proportion of genes showing higher scores in resistant individuals suggests that COPD resistance may be a separate variation-conferred phenotype, as opposed to solely reflecting the absence of COPD-predisposition variants.

To determine if the *FCBF* genes are often found together in biological interaction and regulation networks, we used the induced network modules analysis in ConsensusPathDB [20], an integrative database for gene interaction networks. The vast majority of *FCBF* genes (336 of 368, 91.3 %) were mappable in ConsensusPathDB (FCBF tab in Additional file 2). The induced network modules analysis connects *FCBF* genes/proteins (seed network nodes) into modules using knowledge of different types of interactions (network edges) between these seeds and other intermediate network nodes, such as other genes, chemical ligands, and proteins. A single network is composed of one module or several disconnected modules. In our analysis, we considered binary interactions only, including protein-protein interactions, genetic interactions, biochemical reactions, and gene regulatory interactions. The first step in the analysis induced three modules containing 160 (44 % of our set of 368) interacting seeds (Z-score of intermediate nodes ≥ 20; network1 FCBF tab in Additional file 2). The largest module contained 96 % (154) of the seeds and 50 intermediate nodes; the remaining two modules contained a total of six seeds and two intermediates. Removing low confidence interactions (IntScore <0.5) [21] maintained 138 (38 %) seeds (network2 FCBF tab in Additional file 2; one module of 37 intermediate nodes and 126 seeds, plus five smaller networks encompassing the remaining 12 seeds and 2 intermediates). Building a network of solely high-confidence (IntScore >0.95) protein-protein and biochemical interactions forms a module of 111 (30 %) connected seeds (network 3 FCBF tab in Additional file 2; 24 intermediate nodes, 96 seeds in a single network, plus six smaller modules with 15 seeds and 3 intermediates). Finally, leaving only high-confidence protein-protein interactions retains 43 (12 %) interacting seeds (network 4 FCBF tab in Additional file 2; 12 intermediates and 20 seeds in a single module, plus nine smaller modules of 23 seeds and 7 intermediates, total). Eliminating all intermediates resulted in a high-confidence module of 8-seed proteins (network 5 FCBF tab in Additional file 2). Mutagenesis of interacting proteins without a link to a common phenotype is an unlikely event. Thus, these results suggest that, in the absence of otherwise unifying features of tested individuals, a disruption of an as yet undescribed molecular pathway may be involved in generating the COPD phenotype.

### Candidate gene filtering using RNA-Seq in vitro analysis

Both bronchial and alveolar epithelial cells are the primary target of CS exposure. To determine the abundance of gene expression in primary human bronchial epithelial cells (pHBECs), we conducted RNA-Seq using pHBEC cultures isolated from five nonsmoking donors. We found that 96.2 % of all genes are expressed within a tenfold difference among the five donors. Therefore, we prioritized the candidate genes expressing at an average fragments per kilobase of transcript per million mapped reads (FPKM) of 1.0 or more (50.4 % of 32,457 transcripts) in pHBECs, assuming that transcripts with an FPKM value below 1.0 have low expression [22] (Additional file 3).

Three sets of candidate genes were screened for their expression levels: genes from the (1) VAAST analysis (Additional file 1) and the (2) gene functional deficit score analysis using the *t* test (TTest tab in Additional file 2) and (3) FCBF (FCBF tab in Additional file 2). For the VAAST analysis, we first filtered candidate genes using the following criteria: (1) present in both dominant and recessive analyses under the 5 % background allele frequency setting (i.e., present in analyses 1 and 5 or in analyses 2 and 6 in Table 2) and (2) having a gene ranking <300 in at least one of the analyses. These selection criteria resulted in 363 candidate genes from VAAST analysis. For the TTest and FCBF analyses, we included all candidate genes. Among the three sets of candidate genes, 363, 498, and 199 genes have an average FPKM value larger than 1.0, including 932 unique genes. To generate a set of candidate genes for in vitro functional analysis, we further examined the biological relevance of these candidate genes based on current knowledge of COPD. In the end, we selected a total of 81 of the highest scoring candidate genes for siRNA screening from (1) VAAST analysis (*n* = 42) and (2) FCBF and/or *t* test analysis (*n* = 45). Six genes overlapped between the VAAST and FCBF or *t* test analyses (Additional file 4). All variants with annotations within the 81 candidate genes are listed in Additional file 5.

To confirm these genes are relevant to COPD, we performed Gene Ontology (GO) term and pathway enrichment analysis on the 81 candidate genes. Among the 81 genes, a number of GO terms showed significant enrichment (Additional file 6). The top genes are associated with cell death, which supports our hypothesis that the COPD susceptible genes are associated with cell viability and response to cigarette smoke extract (CSE) toxicity. In addition, using the induced network modules analysis in ConsensusPathDB, 48 out of the 81 genes are connected in a network when allowing intermediate nodes, supporting their involvement in a common pathway (data not shown). Note that since the candidate genes were selected from the computationally identified gene set using expert knowledge, this enrichment in relevant GO terms does not indicate discovery but rather confirms our findings.

### siRNA screening in vitro analysis

**Fig. 2:**
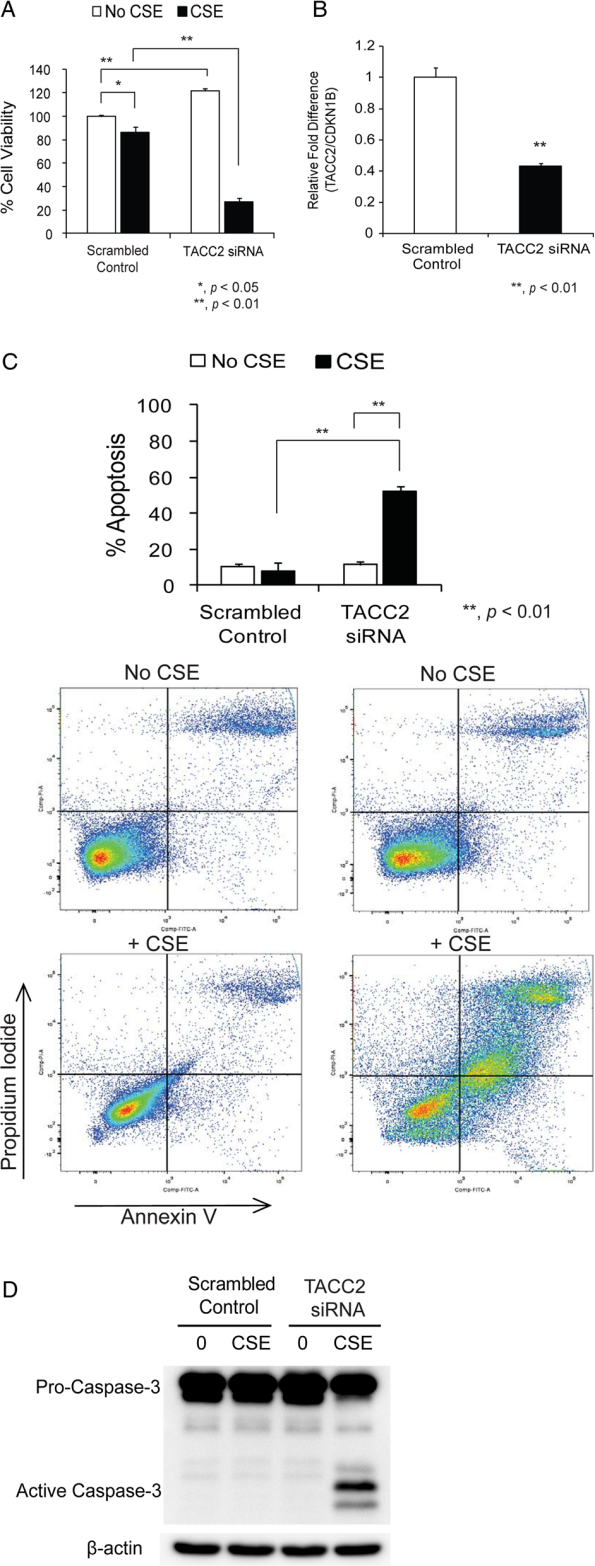
Effects of TACC2 siRNA transfection on CSE-induced cytotoxicity and *TACC2* mRNA levels. **a** Forty-eight hours after transfection with either siRNA targeting *TACC2* (TACC2 siRNA) or the scrambled siRNA (scrambled control) as control, HBEC2 cells were incubated in the absence (no CSE) or presence of 2 % CSE (CSE) for 24 h. Cell viability was determined using the MTT assay at 24 h. Data are expressed as mean ± SEM for three independent experiments with triplicated samples (*p < 0.05; **p < 0.01). **b** Steady-state levels of *TACC2* mRNA were measured by RT-PCR and presented as relative fold difference compared with *CDKN1B* in HBEC2 cells after 48 h with either TACC2 siRNA or scrambled control. Data are expressed as mean ± SEM from two independent experiments with triplicated samples (**p < 0.01). **c** HBEC2 cells were treated as in **a**. Cell death was analyzed by Annexin V and propidium iodide (PI) staining 24 h after 2 % CSE exposure. The percentage of Annexin V positive cells/total cell number was expressed as percentage apoptosis. Data are expressed as mean ± SEM for three independent experiments (**p < 0.01). Representative flow cytometry data are shown. **d** HBEC2 cells were treated as in **a**. Immunoblot analysis of active caspase-3 was performed 24 h after 2 % CSE exposure. Immunoblotting data are representative of three experiments

To determine whether the selected candidate genes (*n* = 81) are important for cell survival against cigarette smoke exposure, we examined the effects of 2 % CSE on cell viability in cultured HBECs (HBEC2) in which the individual gene expression had been suppressed using siRNA (Additional file 4). According to the gene silencing-CSE interaction analysis, we identified two candidate genes whose gene silencing augmented CSE-induced cytotoxicity (*TACC2* and *MYO1E*) and one candidate gene whose gene silencing protected against CSE-induced cytotoxicity (*SLC7A1*) (Tables 3 and 4). Among the candidate genes, suppression of *TACC2* expression induced the most pronounced effect of CSE on cell viability (Table 3 and Additional file 4). We confirmed the effects of siRNA transfection on CSE-induced cytotoxicity (Fig. 2a) and gene silencing by RT-PCR (Fig. 2b). We also performed flow cytometric analysis with Annexin V and propidum iodide staining to characterize the CSE-induced cytotoxicity in HBEC2 cells transfected with TACC2 siRNA. The percentage of early and late apoptotic cells in response to CSE was significantly increased by *TACC2* knockdown (52.2 ± 11.6 %) as compared to scrambled controls (8.1 ± 1.7 %) (Fig. 2c). Furthermore, *TACC2* knockdown activated caspase-3 in CSE-exposed HBEC2 (Fig. 2d). These data suggest that the effects of CSE on cytotoxicity of *TACC2* knockdown cells may largely be due to apoptosis.

**Table 3:** Top five candidate genes whose gene silencing augmented CSE-induced cytotoxicity

	Gene ID	Interaction (AD/CB)	Z-score	*p* value	BH FDR
1	TACC2*	0.196	−6.090	0.0000	0.0010
2	MYO1E*	0.614	−2.924	0.0017	0.0020
3	*NPLOC4*	0.727	−2.070	0.0192	0.0030
4	*FUT2*	0.763	−1.796	0.0363	0.0040
5	*PEX26*	0.797	−1.537	0.0622	0.0050

*p value < BH FDR

**Table 4:** Top five candidate genes whose gene silencing protected against CSE-induced cytotoxicity

	Gene ID	Interaction (AD/CB)	Z-score	*p* value	BH FDR
1	SLC7A1*	1.334	2.959	0.0015	0.0016
2	*INO80*	1.213	1.882	0.0300	0.0032
3	*AXIN1*	1.190	1.878	0.0302	0.0048
4	*EREG*	1.189	1.673	0.0471	0.0065
5	*MAP3K10*	1.177	1.519	0.0644	0.0081

*p value < BH FDR

We also identified three candidate genes whose suppression alone (in the absence of CSE) significantly reduced cell viability (*PLCH2*, *KIAA1919*, and *MRPS34*) (Table 5). These results demonstrate that WES can identify candidate genes whose gene silencing significantly alters spontaneous or CSE-induced cytotoxicity.

**Table 5:** Top five candidate genes whose gene silencing alone reduced cell viability

	Gene ID	Ratio (C/A)	Z-score	*p* value	BH FDR
1	PLCH2*	0.490	−3.359	0.00039	0.00109
2	KIAA1919*	0.492	−3.342	0.00041	0.00217
3	MRPS34*	0.528	−3.030	0.00122	0.00326
4	*EPPK1*	0.607	−2.365	0.00901	0.00435
5	*NAT10*	0.612	−2.281	0.01127	0.00544

*p value < BH FDR

## Discussion

We conducted a WES study using an extreme phenotype design (susceptible vs. resistant smokers) to prioritize candidate genes that potentially modify COPD susceptibility. Among the prioritized candidate genes expressed in pHBECs, we identified two genes (*TACC2* and *MYO1E*) that exhibited a strong interaction of gene (siRNA transfection) with environment (CS exposure) on cytotoxicity in vitro.

Missing heritability is a well-recognized phenomenon in GWAS of complex phenotypes [23, 24]. Potential explanations for missing heritability include epistatic interactions (GxG) and gene-environment interactions (GxE), as well as experimental limitations, e.g. discarding informative variants due to stringent GWAS significance thresholds and insufficient linkage disequilibrium between typed and “causal” variants. Rare disease-causing variants may also be responsible for a substantial proportion of the missing heritability [25–27].

To identify candidate genes from the WES dataset, we used two complementary approaches that focused on either rare variants with presumably large effect sizes (VAAST analyses) or on multiple weakly deleterious variants causing molecular function changes (gene functional deficit score-based TTest and FCBF analyses). We used these diverse approaches because the genetic model for our phenotypes of interest is largely unknown. For example, if COPD is caused by the accumulation of multiple mutations that weakly alter the function of several genes in a certain molecular pathway, the VAAST approach is unlikely to identify the underlying causes and the gene deficit analysis is more appropriate. Unfortunately, the hypothesis of multiple interacting mutations giving rise to a phenotype is very challenging to test with both in vitro and in vivo models, as simultaneous evaluation of multiple mutations is necessary. Nevertheless, we expect that mapping these candidate genes will identify crucial molecular pathways responsible for the development of the resistance and COPD phenotypes.

The current state of the art, as described in the scientific literature, confirms some genes identified by our analysis: of the complete set of *FCBF* genes, 32 were found by a computational parsing of articles identified by the MeSH term “pulmonary disease, chronic obstructive”—the explicit term referring to COPD; another 20 genes were identified by searching for COPD related keywords (chronic obstructive lung disease, chronic obstructive airway disease, chronic airflow limitation, chronic obstructive respiratory disease, chronic bronchitis, COAD, COPD) (FCBF tab in Additional file 2). These results suggest that the selected gene set contains a substantial number of known COPD genes and is possibly enriched in yet undescribed COPD and/or COPD-resistance associated genes. With this knowledge, we will be able to examine the role of gene-gene interaction and weak functionally deleterious mutations in the future as larger sample sizes for genetic studies are acquired and more sophisticated genome-editing technologies are employed in functional studies.

A total of 81 candidate COPD genes selected from our analysis were functionally tested using an in vitro gene knockdown model combined with exposure to CSE. We chose the 3-(4,5-dimethythiazol-2-yl)-2,5-diphenyl tetrazolium bromide (MTT) assay as a measure of cell viability because it is more sensitive in detecting CSE effects (such as cell growth inhibition) than the trypan blue exclusion or LDH release assay (unpublished data). Notably, in addition to cigarette smoking, premature growth of the lung is also thought to be a risk factor for COPD [28]. Therefore, when analyzing the siRNA screening data, we considered two experimental states: (1) gene-environment interaction (both gene silencing and CSE exposure) and (2) siRNA-induced cytotoxicity (gene silencing alone). From the interaction analysis, we identified two candidate genes (*TACC2* and *MYO1E*) whose knockdown increased CSE-induced cytotoxicity. Both genes were identified in susceptible smokers. *TACC2* gene knockdown most dramatically augmented CSE-induced cytotoxicity. *TACC2*, a member of the transforming acidic coiled-coil-containing protein family, is involved in the regulation of centrosome and microtubule dynamics during cell cycling [29] but is not necessary for cell growth and mouse fertility and development [30]. The *TACC2* functions suggest that it may modulate both resistance (through a gain-of-function mutation) and susceptibility (through a loss-of-function mutation) to smoking-induced COPD. In fact, a recent human study identified a common *TACC2* variant as significantly associated with the resistant smoker phenotype [13]. *MYO1E* encodes a non-muscle class I myosin involved in cytoskeleton dynamics; homozygous non-synonymous SNPs in *MYO1E* are associated with familial focal segmental glomerulosclerosis [31]. A recent in vitro study demonstrated that genetic deletion of *MYO1E* augments LPS-induced chemokine (C-C motif) ligand 2 secretion in inflammatory cells [32]. The effects of CS on the lungs of *Myo1e*^−/−^mice are unknown, but CS exposure may enhance lung inflammation and possibly COPD in *Myo1e*^−/−^mice.

We also identified three candidate genes (*PLCH2*, *KIAA1919*, and *MRPS34*), which significantly augmented cytotoxicity by siRNA knockdown alone. PLCH2 is a member of the phospholipase C superfamily of enzymes that regulate phosphoinositide turnover. *Plch2*^−/−^ mice exhibit no obvious phenotype. However, effects on lung development have not been investigated [33]. *KIAA1919* and *MRPS34* encode a sodium-dependent glucose transporter [34] and mitochondrial ribosomal protein S34 [35], respectively. Future studies will be needed to determine whether these genes are involved in the development of respiratory systems using in vivo animal models.

Our in vitro siRNA screening further revealed a candidate gene whose knockdown protected against CSE-induced cytotoxicity (*SLC7A1*). *SLC7A1* was initially selected from the VAAST analysis of susceptible smokers. Since most rare disease-causing variants represent loss-of-function mutations, and *SLC7A1* was identified in susceptible smokers, we might expect knockdown of this gene to augment CSE-induced cytotoxicity. However, the opposite effect was observed in the siRNA screening. It may be that the specific mutations identified by the VAAST analysis were rare gain-of-function mutations. Further experiments examining the precise mutations are needed.

There are several limitations of this siRNA screening approach, including inability to model gain-of-function mutations or to assess epistasis, possible off-target effects, and considerable variability among the individual siRNA effects on gene silencing (e.g., 2 vs. 20 % of the scrambled control). Further studies using primary murine tracheobronchial cells are required to validate our in vitro findings. There are also other limitations when using a simplistic siRNA/cell viability assay to assess candidate genes. For example, inflammatory and endothelial cells were not evaluated, though both are important in disease progression [36, 37]. A number of genes are known to have high expression in inflammatory cells, such as alveolar macrophages and pulmonary endothelial cells, but not in airway epithelial cells [38, 39]. Based on our requirement that genes be expressed in HBECs, we may have excluded genes that may be important in COPD through mechanisms involving inflammatory cells. In future studies, we will include candidate genes expressed in inflammatory cells, such as alveolar macrophages or lymphocytes. Also note that knocking down genes is a rather blunt method. Recapitulating precise mutations of interest using genome-editing technologies, followed by in vitro functional studies, is likely much more precise and a future goal of ours.

## Conclusions

In this study, we identified candidate COPD genes that augment spontaneous or CSE-induced cytotoxicity through genomic, transcriptomic analyses, and in vitro siRNA screening. In the future, we will determine the effects of candidate gene deletion on the development of spontaneous and/or CS-induced COPD in animal models.

## Methods

### Study subjects

Samples were collected from a cross-sectional case-control study design, with no therapeutic intervention or longitudinal follow-up information. All research involving human subjects was approved by the authors’ institutional review board (University of Iowa, New Mexico VA Healthcare System (NMVAHCS), Lovelace Respiratory Research Institute (LRRI) protocols: #200612711, e#11-631, and #20031684, respectively). Informed consent was obtained from all study subjects. Blood or whole lung tissue samples were obtained from very advanced COPD patients (*n* = 62) and resistant smokers (*n* = 30) enrolled in the University of Iowa, NMVAHCS, LRRI, or the Lung Tissue Research Consortium (LTRC). Advanced stage COPD was defined as stages 3 or 4 COPD using the GOLD executive summary 2007 (forced expiratory volume in 1 s [FEV1]/forced vital capacity [FVC] <0.70 and percentage of predicted FEV1 <50) [40], except for three smokers who have more than 50 % predicted FEV1 but very low percentage of predicted diffusing capacity for carbon monoxide (DLCO) (<30). We define exceptionally resistant smokers as individuals who smoke at least 35 pack years, are >60 years of age, have normal spirometries, and absence of significant comorbidities, such as non-dermal malignancy or coronary artery disease. All study subjects were of self-reported non-Hispanic white ethnicity. We have excluded subjects with severe lung inflammation due to chronic infection, immunosuppression due to HIV infection, and known genetic diseases involving the lung (such as cystic fibrosis or A1ATD).

### DNA extraction

DNA extraction was performed using the QIAamp Gentra Puregene Blood and Tissue kit according to manufacturer’s instructions (Qiagen, Hilden, Germany). For isolation of DNA from lung tissues, cell lysis solution was added to 50 mg of minced lung tissues and then followed by addition of proteinase K and incubated for 3 h at 55 °C. For isolation of DNA from blood samples, 10 mL of whole blood was resuspended in Puregene Red Blood Cell Lysis Solution to lyse red blood cells; this was followed by incubation at room temperature for 5 min. Cell lysis solution was added to the white blood cell pellet and vortexed vigorously for 10 s. To the completely digested tissue and blood lysates, we added RNase A solution and protein precipitation solution according to manufacturer’s instructions. The supernatant from the previous step was added to a tube containing isopropanol and mixed by inverting gently 50 times; 70 % ethanol was added and inverted several times to wash the DNA. After final centrifugation and removal of supernatant, the tubes were air-dried to remove residual ethanol for 5–10 min. DNA hydration solution was added and vortexed for 5 s and followed by incubation at 65 °C for 1 h to dissolve the DNA. Concentration and purity of the collected DNA were determined using the NanoDrop ND-1000 spectrophotometer.

### Whole exome data acquisition, processing, and variant discovery

The whole exome sequencing library was constructed using the SureSelectXT Human All Exon V4+UTRs kit (Agilent, Santa Clara, CA) and sequenced using an Illumina HiSeq 2000 system. The sequencing was performed to achieve high-quality variant identification: overall ~60 million reads were sequenced for each individual, and an average read depth of at least 50× was achieved for all samples.

The variant discovery followed the Genome Analysis Toolkit (GATK) Best Practices recommendations [41]. Briefly, raw sequences were aligned to the human reference genome (version hg 19) using Burrows-Wheeler Aligner (BWA) [42]. The resulting raw alignments (in binary sequence alignment/map format) were processed by Picard (http://broadinstitute.github.io/picard) to remove PCR duplicates, followed by local realignment around indels and base quality score recalibration using GATK IndelRealigner and BaseRecalibrator respectively [43]. Variant discovery was performed using GATK UnifiedGenotyper using the joint calling function on all samples. Raw variants quality scored were recalibrated using GATK VariantRecalibrator and ApplyRecalibration was used to generate a Variant Quality Score logarithm of odds (VQSLOD). Finally, variants were filtered using the following criteria: (1) VQSLOD ≥2 and (2) read depth (DP) ≥6 in at least 80 % of samples. In addition, any individual genotype with DP < 6 or GQ < 30 was considered a no-call in the final dataset. The final dataset along with annotation by ANNOVAR [44] are available as a supplemental file on our website under Published Data (https://xinglab.genetics.rutgers.edu/PublishedData/COPD/AllVariants.zip).

### VAAST Analysis

The variant data were analyzed using the Variant Annotation, Analysis, and Search Tool (VAAST 2.0) package [18]. The variants were annotated for their functional impact using Variant Annotation Tool (VAT) in the VAAST package. Then annotated variants in all susceptible individuals and all resistant individuals were combined into two condenser files using Variant Selection Tool (VST). The susceptible and resistant condenser files were analyzed using VAAST to prioritize candidate genes under both dominant and recessive modes of inheritance, allowing locus heterogeneity. We performed the analysis either assuming complete penetrance of a variant or allowing 5 % prevalence of a variant in the control population. Complete penetrance means that no individual in the background population can carry a certain disease-causing variant, while allowing 5 % prevalence means we estimate the expected disease allele frequency within the background population to be 5 % or lower.

The background population used for the analysis of COPD samples were composed of variants from the 10 Gen dataset [45], 1057 genomes from the 1000 Genome Project Phase I [46], 184 Danish exomes [47], 54 whole genomes from the Complete Genomics Diversity Panel [48], and resistant smoker sample exomes. The background files used for resistant smoker analysis are composed of all the genomes or exomes mentioned above (except the resistant smoker sample exomes) and COPD susceptible sample exomes. We did not include 6500 NHLBI exomes in the COPD sample background because COPD subjects are present in this data set [12].

VAAST candidate-gene prioritization was performed and variants in each gene were scored as a group. A rank list of candidate genes was generated based on their disease-causing probability. The significance level was assessed with individual permutation tests. VAAST analysis parameters used: “-m lrt -lh y --significance 1e-4 -d 1e5 [-r 0.05|-pnt c] -iht [d|r]”.

### Gene functional deficit score-based TTest and FCBF analysis

For the 92 individuals in our cohort, we extracted the set of ANNOVAR annotated exonic variants [44]. We mapped the RefSeq messenger RNA (mRNA) IDs identified by ANNOVAR to UniProt proteins. If a variant mapped to different RefSeq IDs, all affected UniProt IDs were included into the affected set. However, for each variant only the primary isoform of the protein was considered. For each non-synonymous variant, we computed the raw SNAP (screening for non-acceptable polymorphisms) score (range −100 to 100, any score ≥0 is classified as neutral/no functional change, and non-neutral otherwise) [49]. For each gene in every individual, we computed a *gene functional deficit score* as a sum over all gene-specific variant scores. An individual variant score was computed for each: (1) InDel or Stop loss/gain, score = 1, (2) SNAP identified non-neutral non-synonymous variant, score = SNAP score/100, (3) SNAP identified neutral variant score = 0.055, and (4) synonymous variant, score = 0.05. Further, individual variant scores of heterozygous variants were multiplied by a factor of 0.25 to approximately modulate the effects of heterozygosity. Gene scores computed in this fashion are 0 only for genes that have no variants at all. However, further comparison between gene scores for different genes is not possible, as the score is highly dependent on gene length and overall tolerance for variability (e.g., longer genes with more variable regions will tend to score higher while remaining relatively functional biochemically). Note that higher gene scores of statistically significant genes in resistant individuals in our study may indicate genes relevant for a resistance phenotype. We compared the distribution of gene scores for resistant smokers vs. the COPD-affected individuals using the *t* test metric. We also applied to the entire set of genes in leave one out cross-validation (92-fold) the fast correlation-based filter solution (FCBF) feature selection algorithm [50]. The symmetrical uncertainty attribute set evaluation in leave one out cross-validation (WEKA implementation [51], parameters = weka.attributeSelection.SymmetricalUncertAttributeSetEval -s “weka.attributeSelection.FCBFSearch -D false -T −1.7976931348623157E308 -N −1”) was also combined to select a set of genes responsible for the observed phenotype as a group, as opposed to a set of genes individually contributing to the phenotype. The feature set evaluation algorithm [50] evaluates the worth of a set of attributes by measuring its symmetrical uncertainty with respect to another set of attributes. Performance of a particular attribute set was measured here by training J48 tree classifier [52] (parameters: weka.classifiers.meta.AttributeSelectedClassifier -E “weka.attributeSelection.SymmetricalUncertAttributeSetEval ” -S “weka.attributeSelection.FCBFSearch -D false -T −1.7976931348623157E308 -N −1” -W weka.classifiers.trees.J48 -- -C 0.25 -M 2).

### Sanger sequencing validation of variant calls

Sanger sequencing was used to validate the seven variants in *TACC2* genes found from the WES. Primer pairs used to amplify PCR products harboring individual variants were designed using Primer3 (http://biotools.umassmed.edu/bioapps/primer3_www.cgi). The detail information of primers used for sequencing, including the sequences, PCR product sizes, and annealing temperatures, are listed (Additional file 7: Table S1). PCR with those primer pairs was performed in 25-μl reaction with OneTaq DNA polymerase (New England Biolabs, Ipswich, MA). Half of the amount of each reaction was used for gel electrophoresis to confirm the presence of a single amplicon with the expected fragment size. After the confirmation, the remaining PCR product was purified by using Illustra ExoProStar according to the manufacturer’s protocol (GE Healthcare Life Sciences, Buckinghamshire, UK). The purified PCR products were then used for Sanger sequencing (GenScript, Piscataway, NJ).

### Cell culture

Primary HBECs (pHBECs) from human lung tissues were isolated from five nonsmoking donors and were cultured in conventional systems under a protocol approved by the LRRI Institutional Review Board as previously described [53]. HBEC2 cells (immortalized HBECs) were originally generated by Ramirez, et al*.* [54] and maintained as previously described [55]. Experiments were performed in 12-well Costar tissue culture plates at a starting cell density of 15 × 10^3^/cm^2^. Cell counts were performed by an electric particle counter (Beckman Coulter, Indianapolis, IN).

### Preparation of cigarette smoke extract (CSE)

One hundred millimeter research cigarettes (3R4F) were purchased from the University of Kentucky. CSE were prepared as previously described [56].

### RNA-Seq

Total RNA was extracted using trizol from pHBEC cultures of five non-smokers without COPD as previously described [53]. Sequence libraries were constructed using the Illumina TruSeq RNA prep kit (San Diego, CA). Multiplex sequencing was performed using the Illumina HiSeq 2000 system. We generated between 30 and 35 million 2 × 50 pair-end reads per sample. The raw sequences were aligned to the NCBI human genome reference build 37 using BWA, and the expression level of each transcript was determined using Tophat [57]. Gene expressions of approximately 32,500 gene transcripts, including multiple transcript variants, were analyzed (as the fragments per kilobase of transcript per million mapped reads (FPKM)). In total, 96.2 % of the gene transcripts have less than tenfold variability (the interquartile range of log10 expression equal to or less than 1.0). To control the quality of our computation of interquartile range, we have excluded all gene transcripts with 2 or more zero FPKM values among the five subjects.

### Cell viability

Cell viability was determined by measuring the reduction of 3-(4,5-dimethythiazol-2-yl)-2,5-diphenyl tetrazolium bromide (MTT) as previously described [55]. The MTT absorbance was read at 570 nm.

### RNA interference

Small interfering RNA (siRNA) for selected genes and the scrambled (control) siRNA were purchased from Applied Biosystems (Carlsbad, CA). Transfection of siRNA was performed using INTERFERin (Polyplus-tranfection Inc, New York, NY) according to the manufacturer’s instructions. The targeted sequences for the selected genes are available (Additional file 8: Table S2). Twenty-four hours after plating, cells were transfected with the individual siRNA or the scrambled control for 48 h. The transfected cells were exposed to 2 % CSE for 24 h in the absence or presence of CSE as determined by the MTT assay.

### Flow cytometry

To detect apoptosis, flow cytometry using Annexin V-FITC (BD Biosciences, Franklin Lakes, NJ) and propidium iodide (Sigma, St Louis, MO) was performed as previously described [58]. Early or late apoptosis was defined by Annexin V+/PI– or Annexin V+/PI+ staining, respectively.

### Immunoblotting

Immunoblot analysis was performed for active caspase-3 as previously described [59]. Equivalent loading was verified by stripping the blot and reprobing with antibodies to β-actin.

### Statistical analysis

The MTT absorption values from triplicated samples per group were averaged for the following four groups: (A) CSE-unexposed scrambled control cells, (B) CSE-exposed scrambled control cells, (C) CSE-unexposed siRNA-transfected cells, and (D) CSE-exposed siRNA-transfected cells. Every 2–10 genes per independent experiment were tested by siRNA transfection with a scrambled control. The interaction of CSE and siRNA effects on cell viability is quantified by the multiplicative interaction ratio of averaged MTT absorption values ([D/C] divided by [B/A]). This ratio is expected to be low or high if the targeted gene is protective or permissive for CSE-induced cytotoxicity. The mean and robust estimate of the standard deviation (nonparametric pseudo SD = interquartile range divided by 1.35) of these interactions are used to compute a Z-score. A one-side *p* value for each gene (by one-sample *Z*-test) was applied to detect both tails of genes. The effects of siRNA alone were also quantified by the ratio of C/A and further analyzed to detect other candidate genes among the 45 genes, whose siRNA reduced the MTT value compared with the scrambled control. The Benjamini and Hochberg (BH) false discovery rate (FDR) procedure is applied to the sorted list of *p* values (smallest to largest) using the formula BH (*j*) = *αj*/*n*, where α = 0.05 and j is the index number of the list of *p* values and *n* is the total number of candidate genes. The *p* values, p (*j*), and the corresponding genes are considered significant for all genes whose p (*j*) ≤ BH (j).

## Additional files

Additional file 1:
**COPD candidate genes prioritized by the VAAST analysis.** Results from different VAAST run settings (Table 1) are shown in seperated tabs. The tab list and the column definition are shown in the tab "TableList". (XLSX 665 kb) Additional file 2:
**COPD candidate genes prioritized by the Gene Functional Deficit Score-based TTest and FCBF Analysis.** Results from Gene Functional Deficit Score-based t test, FCBF analysis, and induced network modules analysis are shown in different tabs. (XLSX 384 kb) Additional file 3:
**The abundance of gene expression in primary human epithelial cell cultures using RNA-Seq.** Expression level (FPKM) of 32,457 transcripts from five nonsmoking donors and the average expression level are shown. (XLSX 4.16 mb) Additional file 4:
**siRNA screening in vitro analysis for 81 COPD candidate genes.** For the 81 candidate genes, the detailed gene information, selection method, and annotation are shown in the tab "Gene Selection Methods". The siRNA knockdown results in three trials are shown in the tab "siRNA summary". (XLSX 92.6 kb) Additional file 5:
**Annotated variants for the 81 COPD candidate genes.** Whole exome sequencing variants identified in the 81 COPD candidate genes in the 62 susceptible smokers with COPD (tab "Susceptible") and 30 resistant smokers (tab "Resistant"). The variant list is in VCF format and annotated with ANNOVAR, except the first column which lists the gene id. (XLSX 1.23 mb ) Additional file 6:
**Gene Ontology term enrichment analysis on the 81 candidate genes.** (XLSX 19.0 kb) Additional file 7: Table S1. The information of Sanger sequencing validation for seven exonic variants of TACC2. Additional file 8: Table S2. Table S2 siRNA sequences targeting 81 COPD candidate genes.

## Abbreviations

A1ATD: alpha1 antitrypsin deficiency
BH: Benjamini and Hochberg
BWA: Burrows-Wheeler aligner
COPD: chronic obstructive pulmonary disease
CS: cigarette smoke
CSE: cigarette smoke extract
DP: read depth
FCBF: fast correlation-based filter solution
FDR: false discovery ratio
FEV1: forced expiratory volume in 1 second
FPKM: fragments per kilobase of transcript per million mapped reads
FVC: forced vital capacity
GATK: Genome Analysis Toolkit
GWAS: genome-wide association study
HBECs: human bronchial epithelial cells
MTT: 3-(4,5-dimethythiazol-2-yl)-2,5-diphenyl tetrazolium bromide
pHBECs: primary human bronchial epithelial cells
siRNA: Small interfering RNA
VAAST: Variant Annotation, Analysis, and Search Tool
VAT: Variant Annotation Tool
VQSLOD: variant quality score logarithm of odds
VST: Variant Selection Tool
WES: whole exome sequencing

## References

[1] Vos T, Flaxman AD, Naghavi M, Lozano R, Michaud C, Ezzati M (2012). Years lived with disability (YLDs) for 1160 sequelae of 289 diseases and injuries 1990–2010: a systematic analysis for the Global Burden of Disease Study 2010. Lancet.

[2] Lozano R, Naghavi M, Foreman K, Lim S, Shibuya K, Aboyans V (2012). Global and regional mortality from 235 causes of death for 20 age groups in 1990 and 2010: a systematic analysis for the Global Burden of Disease Study 2010. Lancet.

[3] Lokke A, Lange P, Scharling H, Fabricius P, Vestbo J (2006). Developing COPD: a 25 year follow up study of the general population. Thorax.

[4] Silverman EK (2006). Progress in chronic obstructive pulmonary disease genetics. Proc Am Thorac Soc.

[5] Janus ED, Phillips NT, Carrell RW (1985). Smoking, lung function, and alpha 1-antitrypsin deficiency. Lancet.

[6] Thun GA, Ferrarotti I, Imboden M, Rochat T, Gerbase M, Kronenberg F (2012). SERPINA1 PiZ and PiS heterozygotes and lung function decline in the SAPALDIA cohort. PLoS One.

[7] American Thoracic Society/European Respiratory Society statement: standards for the diagnosis and management of individuals with alpha-1 antitrypsin deficiency. Am J Respir Crit Care Med. 2003;168 (7):818–900. doi:10.1164/rccm.168.7.818 10.1164/rccm.168.7.818 14522813

[8] Cho MH, Boutaoui N, Klanderman BJ, Sylvia JS, Ziniti JP, Hersh CP (2010). Variants in FAM13A are associated with chronic obstructive pulmonary disease. Nat Genet.

[9] Wilk JB, Shrine NR, Loehr LR, Zhao JH, Manichaikul A, Lopez LM (2012). Genome-wide association studies identify CHRNA5/3 and HTR4 in the development of airflow obstruction. Am J Respir Crit Care Med.

[10] Cho MH, Castaldi PJ, Wan ES, Siedlinski M, Hersh CP, Demeo DL (2012). A genome-wide association study of COPD identifies a susceptibility locus on chromosome 19q13. Hum Mol Genet.

[11] Hersh CP, Pillai SG, Zhu G, Lomas DA, Bakke P, Gulsvik A (2010). Multistudy fine mapping of chromosome 2q identifies XRCC5 as a chronic obstructive pulmonary disease susceptibility gene. Am J Respir Crit Care Med.

[12] Fu W, O’Connor TD, Jun G, Kang HM, Abecasis G, Leal SM, Gabriel S, Rieder MJ, Altshuler D, Shendure J, Nickerson DA, Bamshad MJ, Project NES, Akey JM (2013). Analysis of 6,515 exomes reveals the recent origin of most human protein-coding variants. Nature.

[13] Wain LV, Sayers I, Soler Artigas M, Portelli MA, Zeggini E, Obeidat M (2014). Whole exome re-sequencing implicates CCDC38 and cilia structure and function in resistance to smoking related airflow obstruction. PLoS Genet.

[14] Barnett IJ, Lee S, Lin X (2013). Detecting rare variant effects using extreme phenotype sampling in sequencing association studies. Genet Epidemiol.

[15] Nyunoya T, Mebratu Y, Contreras A, Delgado M, Chand HS, Tesfaigzi Y (2014). Molecular processes that drive cigarette smoke-induced epithelial cell fate of the lung. Am J Respir Cell Mol Biol.

[16] Petrache I, Natarajan V, Zhen L, Medler TR, Richter AT, Cho C (2005). Ceramide upregulation causes pulmonary cell apoptosis and emphysema-like disease in mice. Nat Med.

[17] Aoshiba K, Zhou F, Tsuji T, Nagai A. DNA damage as a molecular link in the pathogenesis of COPD in smokers. Eur Respir J. 2012. doi:10.1183/09031936.00050211 10.1183/09031936.00050211 22267761

[18] Hu H, Huff CD, Moore B, Flygare S, Reese MG, Yandell M (2013). VAAST 2.0: improved variant classification and disease-gene identification using a conservation-controlled amino acid substitution matrix. Genet Epidemiol.

[19] Yandell M, Huff C, Hu H, Singleton M, Moore B, Xing J (2011). A probabilistic disease-gene finder for personal genomes. Genome Res.

[20] Kamburov A, Stelzl U, Lehrach H, Herwig R (2013). The ConsensusPathDB interaction database: 2013 update. Nucleic Acids Res.

[21] Kamburov A, Stelzl U, Herwig R (2012). IntScore: a web tool for confidence scoring of biological interactions. Nucleic Acids Res.

[22] Treutlein B, Brownfield DG, Wu AR, Neff NF, Mantalas GL, Espinoza FH (2014). Reconstructing lineage hierarchies of the distal lung epithelium using single-cell RNA-seq. Nature.

[23] Manolio TA, Collins FS, Cox NJ, Goldstein DB, Hindorff LA, Hunter DJ (2009). Finding the missing heritability of complex diseases. Nature.

[24] Eichler EE, Flint J, Gibson G, Kong A, Leal SM, Moore JH (2010). Missing heritability and strategies for finding the underlying causes of complex disease. Nat Rev Genet.

[25] Gibson G (2011). Rare and common variants: twenty arguments. Nat Rev Genet.

[26] Kryukov GV, Pennacchio LA, Sunyaev SR (2007). Most rare missense alleles are deleterious in humans: implications for complex disease and association studies. Am J Hum Genet.

[27] Schork NJ, Murray SS, Frazer KA, Topol EJ (2009). Common vs. rare allele hypotheses for complex diseases. Curr Opin Genet Dev.

[28] Eisner MD, Anthonisen N, Coultas D, Kuenzli N, Perez-Padilla R, Postma D (2010). An official American Thoracic Society public policy statement: novel risk factors and the global burden of chronic obstructive pulmonary disease. Am J Respir Crit Care Med.

[29] Ha GH, Kim JL, Breuer EK (2013). Transforming acidic coiled-coil proteins (TACCs) in human cancer. Cancer Lett.

[30] Schuendeln MM, Piekorz RP, Wichmann C, Lee Y, McKinnon PJ, Boyd K (2004). The centrosomal, putative tumor suppressor protein TACC2 is dispensable for normal development, and deficiency does not lead to cancer. Mol Cell Biol.

[31] Mele C, Iatropoulos P, Donadelli R, Calabria A, Maranta R, Cassis P (2011). MYO1E mutations and childhood familial focal segmental glomerulosclerosis. N Engl J Med.

[32] Wenzel J, Ouderkirk JL, Krendel M, Lang R (2015). Class I myosin Myo1e regulates TLR4-triggered macrophage spreading, chemokine release, and antigen presentation via MHC class II. Eur J Immunol.

[33] Kanemaru K, Nakahara M, Nakamura Y, Hashiguchi Y, Kouchi Z, Yamaguchi H (2010). Phospholipase C-eta2 is highly expressed in the habenula and retina. Gene Expr Patterns.

[34] Horiba N, Masuda S, Takeuchi A, Takeuchi D, Okuda M, Inui K (2003). Cloning and characterization of a novel Na+-dependent glucose transporter (NaGLT1) in rat kidney. J Biol Chem.

[35] Ogawa F, Adachi S, Kohu K, Shige K, Akiyama T (2003). Binding of the human homolog of the Drosophila discs large tumor suppressor protein to the mitochondrial ribosomal protein MRP-S34. Biochem Biophys Res Commun.

[36] Hautamaki RD, Kobayashi DK, Senior RM, Shapiro SD (1997). Requirement for macrophage elastase for cigarette smoke-induced emphysema in mice. Science (New York, NY).

[37] Nakanishi K, Takeda Y, Tetsumoto S, Iwasaki T, Tsujino K, Kuhara H (2011). Involvement of endothelial apoptosis underlying chronic obstructive pulmonary disease-like phenotype in adiponectin-null mice: implications for therapy. Am J Respir Crit Care Med.

[38] Woodruff PG, Koth LL, Yang YH, Rodriguez MW, Favoreto S, Dolganov GM (2005). A distinctive alveolar macrophage activation state induced by cigarette smoking. Am J Respir Crit Care Med.

[39] Carolan BJ, Harvey BG, Hackett NR, O’Connor TP, Cassano PA, Crystal RG (2009). Disparate oxidant gene expression of airway epithelium compared to alveolar macrophages in smokers. Respir Res.

[40] Rabe KF, Hurd S, Anzueto A, Barnes PJ, Buist SA, Calverley P (2007). Global strategy for the diagnosis, management, and prevention of chronic obstructive pulmonary disease: GOLD executive summary. Am J Respir Crit Care Med.

[41] DePristo MA, Banks E, Poplin R, Garimella KV, Maguire JR, Hartl C (2011). A framework for variation discovery and genotyping using next-generation DNA sequencing data. Nat Genet.

[42] Li H, Durbin R (2009). Fast and accurate short read alignment with Burrows-Wheeler transform. Bioinformatics.

[43] McKenna A, Hanna M, Banks E, Sivachenko A, Cibulskis K, Kernytsky A (2010). The Genome Analysis Toolkit: a MapReduce framework for analyzing next-generation DNA sequencing data. Genome Res.

[44] Wang K, Li M, Hakonarson H (2010). ANNOVAR: functional annotation of genetic variants from high-throughput sequencing data. Nucleic Acids Res.

[45] Reese MG, Moore B, Batchelor C, Salas F, Cunningham F, Marth GT (2010). A standard variation file format for human genome sequences. Genome Biol.

[46] Genomes Project C, Abecasis GR, Altshuler D, Auton A, Brooks LD, Durbin RM, Gibbs RA, Hurles ME, McVean GA (2010). A map of human genome variation from population-scale sequencing. Nature.

[47] Li Y, Vinckenbosch N, Tian G, Huerta-Sanchez E, Jiang T, Jiang H (2010). Resequencing of 200 human exomes identifies an excess of low-frequency non-synonymous coding variants. Nat Genet.

[48] Drmanac R, Sparks AB, Callow MJ, Halpern AL, Burns NL, Kermani BG (2010). Human genome sequencing using unchained base reads on self-assembling DNA nanoarrays. Science.

[49] Bromberg Y, Rost B (2007). SNAP: predict effect of non-synonymous polymorphisms on function. Nucleic Acids Res.

[50] Yu L, Liu H (2003). Feature selection for high-dimensional data: a fast correlation-based filter solution. Proceedings of the twentieth international conference on machine learning.

[51] Hall M, Frank E, Holmes G, Pfahringer B, Reutemann P, Wittn I (2009). The WEKA data mining software: an update. ACM SIGKDD Explor Newsl.

[52] Quinlan JR (1993). Book Review: C4.5: programs for machine learning. Mach Learn.

[53] Jang JH, Bruse S, Liu Y, Duffy V, Zhang C, Oyamada N (2014). Aldehyde dehydrogenase 3A1 protects airway epithelial cells from cigarette smoke-induced DNA damage and cytotoxicity. Free Radic Biol Med.

[54] Ramirez RD, Sheridan S, Girard L, Sato M, Kim Y, Pollack J (2004). Immortalization of human bronchial epithelial cells in the absence of viral oncoproteins. Cancer Res.

[55] Jang JH, Bruse S, Liu Y, Duffy V, Zhang C, Oyamada N (2013). Aldehyde dehydrogenase 3A1 protects airway epithelial cells from cigarette smoke-induced DNA damage and cytotoxicity. Free Radic Biol Med.

[56] Nyunoya T, Monick MM, Klingelhutz AL, Glaser H, Cagley JR, Brown CO (2009). Cigarette smoke induces cellular senescence via Werner’s syndrome protein down-regulation. Am J Respir Crit Care Med.

[57] Trapnell C, Roberts A, Goff L, Pertea G, Kim D, Kelley DR (2012). Differential gene and transcript expression analysis of RNA-seq experiments with TopHat and Cufflinks. Nat Protoc.

[58] Chand HS, Montano G, Huang X, Randell SH, Mebratu Y, Petersen H (2014). A genetic variant of p53 restricts the mucous secretory phenotype by regulating SPDEF and Bcl-2 expression. Nat Commun.

[59] Shi S, Wang Q, Xu J, Jang JH, Padilla MT, Nyunoya T (2015). Synergistic anticancer effect of cisplatin and Chal-24 combination through IAP and c-FLIPL degradation, Ripoptosome formation and autophagy-mediated apoptosis. Oncotarget.

