# Correction to: Whole exome sequencing identifies novel candidate genes that modify chronic obstructive pulmonary disease susceptibility

**DOI:** 10.1186/s40246-021-00373-w

**Published:** 2021-12-30

**Authors:** Shannon Bruse, Michael Moreau, Yana Bromberg, Jun-Ho Jang, Nan Wang, Hongseok Ha, Maria Picchi, Yong Lin, Raymond J. Langley, Clifford Qualls, Julia Klesney-Tait, Joseph Zabner, Shuguang Leng, Jenny Mao, Steven A. Belinsky, Jinchuan Xing, Toru Nyunoya

**Affiliations:** 1 Lovelace Respiratory Research Institute, 2425 Ridgecrest Drive SE, Albuquerque, NM 87108 USA; 2 Department of Genetics, Rutgers, The State University of New Jersey, 145 Bevier Road, Piscataway, NJ 08854 USA; 3 Department of Biochemistry and Microbiology, Rutgers, The State University of New Jersey, Piscataway, NJ USA; 4 Department of Internal Medicine, University of New Mexico and New Mexico VA Health Care System, Albuquerque, NM USA; 5 Biomedical Research Institute of New Mexico, Albuquerque, NM USA; 6 Department of Medicine, University of Iowa, Iowa City, IA USA

## Correction to: Human Genomics (2016) 10:1 10.1186/s40246-015-0058-7

Following the publication of the original article [1], the authors identified an error in the author names of Julia Klesney-Tait.

The incorrect author name is: Julia Klensney-Tait.

The correct author names is: Julia Klesney-Tait.

The original article [1] has been updated.
