# Supplementary material for: Whole exome sequencing identifies novel candidate genes that modify chronic obstructive pulmonary disease susceptibility
Source: Hum Genomics. 2016 Jan 7;10:1. doi: 10.1186/s40246-015-0058-7 (PMC4705629; doi:10.1186/s40246-015-0058-7)
Supplement: Additional file 7: Table S1. — The information of Sanger sequencing validation for seven exonic variants of TACC2. [file 40246_2015_58_MOESM7_ESM.docx]

Supplementary Table S1. The information of Sanger sequencing validation for seven exonic variants of *TACC2*

| **Location** | **Position** | **Primer-F** | **Primer-R** | **T_a_**  **(°C)** | **Size (bp)** | **Sample** | **Genotype** | **Sample** | **Genotype** |
| --- | --- | --- | --- | --- | --- | --- | --- | --- | --- |
| TACC2_1 | chr10:123842508 | CCTTGGCATCCATGATAACC | GTACTGGCGACATTCCAGGT | 55 | 507 | 30 | C/T^a^ |  |  |
| TACC2_3 | chr10:123844900 | GACTTCTCCAAGCCATCCAG | CAACCATCAGCTGCTTCAGA | 55 | 501 | 44 | C/T^a^ |  |  |
| TACC2_4 | chr10:123903149 | AGAAGTGGAGGCCTGGATG | CATGCTTCCCACTGTGACAT | 55 | 510 | 14 | G/A^b^ |  |  |
| TACC2_6 | chr10:123970638 | AGACCACCAAGAAACCCACA | TTCATCTTTGGCCTCCTCAG | 55 | 499 | 19 | C/T^a^ |  |  |
| TACC2_7 | chr10:123987443 | CTTCCCCCAGGTCAAGTTTT | GGCAGAGGACTGCTTAACGA | 55 | 498 | 26 | C/T^a^ |  |  |
| TACC2_8 | chr10:123996970 | AATAGTGCCCTGGGTTCTCA | CACATGCCACGATACCTCTG | 60 | 501 | 61 | T/C^b^ |  |  |
| TACC2_10 | chr10:124009124 | ATCAGTGGAGGATGCGTGTT | GCTGGCATGAAATTGGAACT | 60 | 512 | 32 | C/C^a^ | 14 | G/C^a^ |
|  |  |  |  |  |  |  |  |  |  |

These variants were validated by Sanger sequencing with **^a^** both forward and reverse primers; or **^b^** reverse primer.
