# Supplementary material for: Whole exome sequencing identifies novel candidate genes that modify chronic obstructive pulmonary disease susceptibility
Source: Hum Genomics. 2016 Jan 7;10:1. doi: 10.1186/s40246-015-0058-7 (PMC4705629; doi:10.1186/s40246-015-0058-7)
Supplement: Additional file 8: Table S2. — Table S2 siRNA sequences targeting 81 COPD candidate genes. [file 40246_2015_58_MOESM8_ESM.docx]

|  | **Gene Name** | **Sense siRNA Sequence** | **Antisense siRNA Sequence** |
| --- | --- | --- | --- |
| AGRN | Agrin | GGCGGACCUUUGUCGAGUAtt | UACUCGACAAAGGUCCGCCcg |
| [AHCTF1](http://www.ncbi.nlm.nih.gov/entrez/query.fcgi?db=gene&cmd=search&term=AHCTF1) | AT hook containing transcription factor 1 | GAAUGAUCGUGAUCCUCGUtt | ACGAGGAUCACGAUCAUUCat |
| [AHCYL1](http://www.ncbi.nlm.nih.gov/entrez/query.fcgi?db=gene&cmd=search&term=AHCYL1) | Adenosylhomocysteinase-like 1 | GGGUGGUAAAGCUAAAUGAtt | UCAUUUAGCUUUACCACCCtg |
| AHSA1 | AHA1, activator of heat shock 90kDa protein ATPase homolog 1 (yeast) | GACAGGUACUUCUAAGUCAtt | UGACUUAGAAGUACCUGUCca |
| [ARL15](http://www.ncbi.nlm.nih.gov/entrez/query.fcgi?db=gene&cmd=search&term=ARL15) | ADP-ribosylation factor-like 15 | GUUAUGCACUUUACCCUUUtt | AAAGGGUAAAGUGCAUAACtg |
| [ARSD](http://www.ncbi.nlm.nih.gov/entrez/query.fcgi?db=gene&cmd=search&term=ARSD) | Arylsulfatase D | GGUUGCUACGGGAACAAUAtt | UAUUGUUCCCGUAGCAACCga |
| AXIN1 | Axin 1 | GGGAUAAGCCUGUUCAGGAtt | UCCUGAACAGGCUUAUCCCat |
| [BSCL2](http://www.ncbi.nlm.nih.gov/entrez/query.fcgi?db=gene&cmd=search&term=BSCL2) | Bernardinelli-Seip congenital lipodystrophy 2 (seipin) | GAACUCUACGCAGACUAUAtt | UAUAGUCUGCGUAGAGUUCca |
| [BTN2A1](http://www.ncbi.nlm.nih.gov/entrez/query.fcgi?db=gene&cmd=search&term=BTN2A1) | Butyrophilin, subfamily 2, member A1 | CCAUUGCCGUAUGCAUCUAtt | UAGAUGCAUACGGCAAUGGgt |
| [BZW1](http://www.ncbi.nlm.nih.gov/entrez/query.fcgi?db=gene&cmd=search&term=BZW1) | Basic leucine zipper and W2 domains 1 | GGUUUUUCAGAGUCGGAGAtt | UCUCCGACUCUGAAAAACCct |
| [C6orf72](http://www.ncbi.nlm.nih.gov/entrez/query.fcgi?db=gene&cmd=search&term=C6orf72) | Chromosome 6 open reading frame 72 | GUGUAAGGAUUUUAGUUCAtt | UGAACUAAAAUCCUUACACtg |
| [CERK](http://www.ncbi.nlm.nih.gov/entrez/query.fcgi?db=gene&cmd=search&term=CERK) | Ceramide kinase | GUCAAAUACCAAUGCUUUAtt | UAAAGCAUUGGUAUUUGACat |
| [CLDN7](http://www.ncbi.nlm.nih.gov/entrez/query.fcgi?db=gene&cmd=search&term=CLDN7) | Claudin 7 | UGAUCCCUACCAACAUUAAtt | UUAAUGUUGGUAGGGAUCAaa |
| FAM36A/[COX20](http://www.ncbi.nlm.nih.gov/entrez/query.fcgi?db=gene&cmd=search&term=COX20) | COX20 cytochrome C oxidase assembly factor | AAUAUUGUAUGGUUCAUUAtt | UAAUGAACCAUACAAUAUUga |
| [CSF2](http://www.ncbi.nlm.nih.gov/entrez/query.fcgi?db=gene&cmd=search&term=CSF2) | Colony stimulating factor 2 (granulocyte-macrophage) | CAACCCAGAUUAUCACCUUtt | AAGGUGAUAAUCUGGGUUGca |
| CTSB | Cathepsin B | AGAGAGUUAUGUUUACCGAtt | UCGGUAAACAUAACUCUCUgg |
| [CYP1B1](http://www.ncbi.nlm.nih.gov/entrez/query.fcgi?db=gene&cmd=search&term=CYP1B1) | Cytochrome P450, family 1, subfamily B, polypeptide 1 | CCCAAGUCAUUUAAAGUCAtt | UGACUUUAAAUGACUUGGGtt |
| [CYR61](http://www.ncbi.nlm.nih.gov/entrez/query.fcgi?db=gene&cmd=search&term=CYR61) | Cysteine-rich, angiogenic inducer, 61 | CCACACGAGUUACCAAUGAtt | UCAUUGGUAACUCGUGUGGag |
| [DEK](http://www.ncbi.nlm.nih.gov/entrez/query.fcgi?db=gene&cmd=search&term=DEK) | DEK oncogene | GAAAACCGAUGAACUUAGAtt | UCUAAGUUCAUCGGUUUUCtt |
| [DHX58](http://www.ncbi.nlm.nih.gov/entrez/query.fcgi?db=gene&cmd=search&term=DHX58) | DEXH (Asp-Glu-X-His) box polypeptide 58 | ACGCGUUUGUAGCAACUGAtt | UCAGUUGCUACAAACGCGUat |
| [DUSP6](http://www.ncbi.nlm.nih.gov/entrez/query.fcgi?db=gene&cmd=search&term=DUSP6) | Dual specificity phosphatase 6 | CGGACACUAUUAUCACUAAtt | UUAGUGAUAAUAGUGUCCGta |
| [DVL2](http://www.ncbi.nlm.nih.gov/entrez/query.fcgi?db=gene&cmd=search&term=DVL2) | Dishevelled, dsh homolog 2 (Drosophila) | CACCAUCCCUAAUGCCUUUtt | AAAGGCAUUAGGGAUGGUGat |
| [EMP1](http://www.ncbi.nlm.nih.gov/entrez/query.fcgi?db=gene&cmd=search&term=EMP1) | Epithelial membrane protein 1 | GUUGGUUUCCAAUACGGUAtt | UACCGUAUUGGAAACCAACca |
| [EPHX1](http://www.ncbi.nlm.nih.gov/entrez/query.fcgi?db=gene&cmd=search&term=EPHX1) | Epoxide hydrolase 1, microsomal (xenobiotic) | GGAUCGAUAAGUUCCGUUUtt | AAACGGAACUUAUCGAUCCtc |
| [EPPK1](http://www.ncbi.nlm.nih.gov/entrez/query.fcgi?db=gene&cmd=search&term=EPPK1) | Epiplakin 1 | CCAGGGUCAUCGAUCAGAAtt | UUCUGAUCGAUGACCCUGGag |
| [EREG](http://www.ncbi.nlm.nih.gov/entrez/query.fcgi?db=gene&cmd=search&term=EREG) | Epiregulin | GCUUUGACCGUGAUUCUUAtt | UAAGAAUCACGGUCAAAGCca |
| [FAM83A](http://www.ncbi.nlm.nih.gov/entrez/query.fcgi?db=gene&cmd=search&term=FAM83A) | Family with sequence similarity 83, member A | GGGCUGACUUUAGUGACAAtt | UUGUCACUAAAGUCAGCCCtg |
| [FUT2](http://www.ncbi.nlm.nih.gov/entrez/query.fcgi?db=gene&cmd=search&term=FUT2) | Fucosyltransferase 2 (secretor status included) | UCUUCGUGGUCACCAGUAAtt | UUACUGGUGACCACGAAGAtg |
| [GALNT5](http://www.ncbi.nlm.nih.gov/entrez/query.fcgi?db=gene&cmd=search&term=GALNT5) | UDP-N-acetyl-alpha-D-galactosamine:polypeptide N-acetylgalactosaminyltransferase 5 (GalNAc-T5) | GGAUAGGAUUCAGAGUUCAtt | UGAACUCUGAAUCCUAUCCgc |
| [GLO1](http://www.ncbi.nlm.nih.gov/entrez/query.fcgi?db=gene&cmd=search&term=GLO1) | Glyoxalase I | ACUGGAUUUUUAUACUAGAtt | UCUAGUAUAAAAAUCCAGUga |
| [HGS](http://www.ncbi.nlm.nih.gov/entrez/query.fcgi?db=gene&cmd=search&term=HGS) | Hepatocyte growth factor-regulated tyrosine kinase substrate | CGUCUUUCCAGAAUUCAAAtt | UUUGAAUUCUGGAAAGACGtg |
| [HIF1A](http://www.ncbi.nlm.nih.gov/entrez/query.fcgi?db=gene&cmd=search&term=HIF1A) | Hypoxia inducible factor 1, alpha subunit (basic helix-loop-helix transcription factor) | CCAUAUAGAGAUACUCAAAtt | UUUGAGUAUCUCUAUAUGGtg |
| [ICAM1](http://www.ncbi.nlm.nih.gov/entrez/query.fcgi?db=gene&cmd=search&term=ICAM1) | Intercellular adhesion molecule 1 | CGGAAGGUGUAUGAACUGAtt | UCAGUUCAUACACCUUCCGgt |
| [INO80](http://www.ncbi.nlm.nih.gov/entrez/query.fcgi?db=gene&cmd=search&term=INO80) | INO80 homolog (S. cerevisiae) | CGGGUACAACGUGUCUAAAtt | UUUAGACACGUUGUACCCGta |
| [KIAA1919](http://www.ncbi.nlm.nih.gov/entrez/query.fcgi?db=gene&cmd=search&term=KIAA1919) | KIAA1919 | GCUCUGUUUGGAGUACCUAtt | UAGGUACUCCAAACAGAGCtt |
| [KLF4](http://www.ncbi.nlm.nih.gov/entrez/query.fcgi?db=gene&cmd=search&term=KLF4) | Kruppel-like factor 4 (gut) | UGACCAGGCACUACCGUAAtt | UUACGGUAGUGCCUGGUCAgt |
| [LAMA1](http://www.ncbi.nlm.nih.gov/entrez/query.fcgi?db=gene&cmd=search&term=LAMA1) | Laminin, alpha 1 | GAUGUUAGCGAGUACCCAAtt | UUGGGUACUCGCUAACAUCct |
| [MAN2B1](http://www.ncbi.nlm.nih.gov/entrez/query.fcgi?db=gene&cmd=search&term=MAN2B1) | Mannosidase, alpha, class 2B, member 1 | CCGUGGACCAGUACUUUUAtt | UAAAAGUACUGGUCCACGGtt |
| [MAP3K10](http://www.ncbi.nlm.nih.gov/entrez/query.fcgi?db=gene&cmd=search&term=MAP3K10) | Mitogen-activated protein kinase kinase kinase 10 | GGCUUUGAGCAUAAGAUCAtt | UGAUCUUAUGCUCAAAGCCag |
| [MPDZ](http://www.ncbi.nlm.nih.gov/entrez/query.fcgi?db=gene&cmd=search&term=MPDZ) | Multiple PDZ domain protein | GGGUAUUGCUAUCAGCGAAtt | UUCGCUGAUAGCAAUACCCaa |
| [MRPS34](http://www.ncbi.nlm.nih.gov/entrez/query.fcgi?db=gene&cmd=search&term=MRPS34) | Mitochondrial ribosomal protein S34 | AACACGUCAUGUACCAUGAtt | UCAUGGUACAUGACGUGUUcg |
| [MYO1E](http://www.ncbi.nlm.nih.gov/entrez/query.fcgi?db=gene&cmd=search&term=MYO1E) | Myosin IE | GGAGCGGCACAGUAUGAAAtt | UUUCAUACUGUGCCGCUCCtt |
| [NADK](http://www.ncbi.nlm.nih.gov/entrez/query.fcgi?db=gene&cmd=search&term=NADK) | NAD kinase | CGUGUAUGUGGAAAAGAAAtt | UUUCUUUUCCACAUACACGat |
| [NAT10](http://www.ncbi.nlm.nih.gov/entrez/query.fcgi?db=gene&cmd=search&term=NAT10) | N-acetyltransferase 10 (GCN5-related) | CAGUGACUAUGGAUGUGCAtt | UGCACAUCCAUAGUCACUGtg |
| [NBAS](http://www.ncbi.nlm.nih.gov/entrez/query.fcgi?db=gene&cmd=search&term=NBAS) | Neuroblastoma amplified sequence | CGAAGCUUGUUUUAACGGUtt | ACCGUUAAAACAAGCUUCGgg |
| [NELF](http://www.ncbi.nlm.nih.gov/entrez/query.fcgi?db=gene&cmd=search&term=NELF) | Nasal embryonic LHRH factor | UGAGCAAAGUGAACCCAGAtt | UCUGGGUUCACUUUGCUCAtc |
| [PNP](http://www.ncbi.nlm.nih.gov/entrez/query.fcgi?db=gene&cmd=search&term=NP) | Nucleoside phosphorylase | CUCAGUACCUGGAAACAAAtt | UUUGUUUCCAGGUACUGAGag |
| [NPLOC4](http://www.ncbi.nlm.nih.gov/entrez/query.fcgi?db=gene&cmd=search&term=NPLOC4) | Nuclear protein localization 4 homolog (S. cerevisiae) | GGACGGGAAGAUUUACAGAtt | UCUGUAAAUCUUCCCGUCCtg |
| [NTPCR](http://www.ncbi.nlm.nih.gov/entrez/query.fcgi?db=gene&cmd=search&term=NTPCR) | Nucleoside-triphosphatase, cancer-related | AAAACAACAUUGAUCCAUAtt | UAUGGAUCAAUGUUGUUUUtc |
| [PDLIM4](http://www.ncbi.nlm.nih.gov/entrez/query.fcgi?db=gene&cmd=search&term=PDLIM4) | PDZ and LIM domain 4 | GCUGAGAACUAAGAGAUGAtt | UCAUCUCUUAGUUCUCAGCta |
| [PEX26](http://www.ncbi.nlm.nih.gov/entrez/query.fcgi?db=gene&cmd=search&term=PEX26) | Peroxisomal biogenesis factor 26 | CCUGGGUCCUUCAGUAUUAtt | UAAUACUGAAGGACCCAGGag |
| [PFKM](http://www.ncbi.nlm.nih.gov/entrez/query.fcgi?db=gene&cmd=search&term=PFKM) | Phosphofructokinase, muscle | GGACUUUCGGGAACGAGAAtt | UUCUCGUUCCCGAAAGUCCtt |
| [PLCH2](http://www.ncbi.nlm.nih.gov/entrez/query.fcgi?db=gene&cmd=search&term=PLCH2) | Phospholipase C, eta 2 | GUAUCUGACUGACAUCCUUtt | AAGGAUGUCAGUCAGAUACtg |
| [POT1](http://www.ncbi.nlm.nih.gov/entrez/query.fcgi?db=gene&cmd=search&term=POT1) | Protection of telomeres 1 | GCUCCUCAACAAUACCGCAtt | UGCGGUAUUGUUGAGGAGCtt |
| [PPID](http://www.ncbi.nlm.nih.gov/entrez/query.fcgi?db=gene&cmd=search&term=PPID) | Peptidylprolyl isomerase D | GCAUGAUCGGGAGGGUUUAtt | UAAACCCUCCCGAUCAUGCtt |
| [PRKCZ](http://www.ncbi.nlm.nih.gov/entrez/query.fcgi?db=gene&cmd=search&term=PRKCZ) | Protein kinase C, zeta | CGUUCGACAUCAUCACCGAtt | UCGGUGAUGAUGUCGAACGgg |
| [RAD51C](http://www.ncbi.nlm.nih.gov/entrez/query.fcgi?db=gene&cmd=search&term=RAD51C) | RAD51 homolog C (S. cerevisiae) | AAGAUAUGCUGGUACAUCUtt | AGAUGUACCAGCAUAUCUUgg |
| RANGAP | Ran GTPase activating protein 1 | GGACUUAAGCGACAACGCAtt | UGCGUUGUCGCUUAAGUCCag |
| [RFWD3](http://www.ncbi.nlm.nih.gov/entrez/query.fcgi?db=gene&cmd=search&term=RFWD3) | Ring finger and WD repeat domain 3 | CCAUUUGAGGUGAACCGUAtt | UACGGUUCACCUCAAAUGGgc |
| [RTN4](http://www.ncbi.nlm.nih.gov/entrez/query.fcgi?db=gene&cmd=search&term=RTN4) | Reticulon 4 | GCCUCUUCUUAGUUGAUGAtt | UCAUCAACUAAGAAGAGGCgc |
| [SERPINB7](http://www.ncbi.nlm.nih.gov/entrez/query.fcgi?db=gene&cmd=search&term=SERPINB7) | Serpin peptidase inhibitor, clade B (ovalbumin), member 7 | GGAUGACAAUCAAGGAAAUtt | AUUUCCUUGAUUGUCAUCCat |
| [SIAH1](http://www.ncbi.nlm.nih.gov/entrez/query.fcgi?db=gene&cmd=search&term=SIAH1) | Siah E3 ubiquitin protein ligase 1 | CGCCCAUUCUUCAAUGUCAtt | UGACAUUGAAGAAUGGGCGgt |
| [SLC7A1](http://www.ncbi.nlm.nih.gov/entrez/query.fcgi?db=gene&cmd=search&term=SLC7A1) | Solute carrier family 7, member 1 | CAUCGGUACUUCAAGCGUAtt | UACGCUUGAAGUACCGAUGat |
| [SNCG](http://www.ncbi.nlm.nih.gov/entrez/query.fcgi?db=gene&cmd=search&term=SNCG) | Synuclein, gamma (breast cancer-specific protein 1) | UCAUGUAUGUGGGAGCCAAtt | UUGGCUCCCACAUACAUGAcc |
| [SNPH](http://www.ncbi.nlm.nih.gov/entrez/query.fcgi?db=gene&cmd=search&term=SNPH) | Syntaphilin | AGACAGACUUCGUGCAGUAtt | UACUGCACGAAGUCUGUCUgg |
| SRCAP | Snf2-related CREBBP activator protein | GGUUGAUGCUAAUAGCUCUtt | AGAGCUAUUAGCAUCAACCtc |
| [TACC2](http://www.ncbi.nlm.nih.gov/entrez/query.fcgi?db=gene&cmd=search&term=TACC2) | Transforming, acidic coiled-coil containing protein 2 | GAGCAGAGAUCAUAACCAAtt | UUGGUUAUGAUCUCUGCUCtg |
| [TANK](http://www.ncbi.nlm.nih.gov/entrez/query.fcgi?db=gene&cmd=search&term=TANK) | TRAF family member-associated NFKB activator | CACUCAAGAUAACAAUUAUtt | AUAAUUGUUAUCUUGAGUGga |
| [TMEM123](http://www.ncbi.nlm.nih.gov/entrez/query.fcgi?db=gene&cmd=search&term=TMEM123) | Transmembrane protein 123 | GCUUCAUCAGUAACAAUCAtt | UGAUUGUUACUGAUGAAGCag |
| [TMEM214](http://www.ncbi.nlm.nih.gov/entrez/query.fcgi?db=gene&cmd=search&term=TMEM214) | Transmembrane protein 214 | CAAACGGAGUGUGGAAAUAtt | UAUUUCCACACUCCGUUUGct |
| [TP53AIP1](http://www.ncbi.nlm.nih.gov/entrez/query.fcgi?db=gene&cmd=search&term=TP53AIP1) | Tumor protein p53 regulated apoptosis inducing protein 1 | UCGGAGCACCAGUCACUUAtt | UAAGUGACUGGUGCUCCGAgg |
| [TP53I11](http://www.ncbi.nlm.nih.gov/entrez/query.fcgi?db=gene&cmd=search&term=TP53I11) | Tumor protein p53 inducible protein 11 | CAUCAGCAUUUACUACUAUtt | AUAGUAGUAAAUGCUGAUGac |
| [TRAPPC10](http://www.ncbi.nlm.nih.gov/entrez/query.fcgi?db=gene&cmd=search&term=TRAPPC10) | Trafficking protein particle complex 10 | GGUUAAUAGUGAUAGUUGAtt | UCAACUAUCACUAUUAACCag |
| [TSPAN4](http://www.ncbi.nlm.nih.gov/entrez/query.fcgi?db=gene&cmd=search&term=TSPAN4) | Tetraspanin 4 | CGGACAAGAUUGACAGGUAtt | UACCUGUCAAUCUUGUCCGtg |
| [USP14](http://www.ncbi.nlm.nih.gov/entrez/query.fcgi?db=gene&cmd=search&term=USP14) | Ubiquitin specific peptidase 14 (tRNA-guanine transglycosylase) | GCAUAUCGCUUACGUUCUAtt | UAGAACGUAAGCGAUAUGCca |
| [USP35](http://www.ncbi.nlm.nih.gov/entrez/query.fcgi?db=gene&cmd=search&term=USP35) | Ubiquitin specific peptidase 35 | ACAACAUCCUUUACCUACAtt | UGUAGGUAAAGGAUGUUGUct |
| [VAV3](http://www.ncbi.nlm.nih.gov/entrez/query.fcgi?db=gene&cmd=search&term=VAV3) | Vav 3 guanine nucleotide exchange factor | GGUCAUUAGGAACUAUUCUtt | AGAAUAGUUCCUAAUGACCtg |
| [WBSCR16](http://www.ncbi.nlm.nih.gov/entrez/query.fcgi?db=gene&cmd=search&term=) | RCC1-like G exchanging factor-like | GGUCCAAACCUAGUGGAAAtt | UUUCCACUAGGUUUGGACCtt |
| [WEE1](http://www.ncbi.nlm.nih.gov/entrez/query.fcgi?db=gene&cmd=search&term=WEE1) | WEE1 homolog (S. pombe) | CAAUUACGAAUAGAAUUGAtt | UCAAUUCUAUUCGUAAUUGtt |
| [ZBTB4](http://www.ncbi.nlm.nih.gov/entrez/query.fcgi?db=gene&cmd=search&term=ZBTB4) | Zinc finger and BTB domain containing 4 | GCUACGCAGUGAAUCCUCAtt | UGAGGAUUCACUGCGUAGCca |
| [ZFAND2B](http://www.ncbi.nlm.nih.gov/entrez/query.fcgi?db=gene&cmd=search&term=ZFAND2B) | Zinc finger, AN1-type domain 2B | CAGCAAAAACGUAAGAUCUtt | AGAUCUUACGUUUUUGCUGtg |
